# Therapeutic Target Mapping to Advance Drug Repurposing for Cardiovascular Disease: A Perspective from an Explanted Heart Biobank

**DOI:** 10.3390/jpm16080416

**Published:** 2026-08-03

**Authors:** Coco Ng, Gurpreet K. Singhera, Ea Chung Chang, Amrit Samra, Katherine A. Adolphs, Evan H. Phillips, Jamil Bashir, Zachary Laksman, Honglin Luo, Gordon A. Francis, Chi Lai, Ying Wang

**Affiliations:** 1 Centre for Heart Lung Innovation, University of British Columbia, Vancouver, BC V6Z 1Y6, Canada; coco.ng@hli.ubc.ca (C.N.); gurpreet.singhera@hli.ubc.ca (G.K.S.); amrit.samra@hli.ubc.ca (A.S.); katherine.adolphs@hli.ubc.ca (K.A.A.); honglin.luo@hli.ubc.ca (H.L.); gordon.francis@hli.ubc.ca (G.A.F.);; 2 Department of Surgery, University of British Columbia, Vancouver, BC V5Z 1M9, Canada; jbashir@providencehealth.bc.ca; 3 St. Paul’s Hospital, Vancouver, BC V6Z 1Y6, Canada; 4 School of Biomedical Engineering, University of British Columbia, Vancouver, BC V6T 2B9, Canada; 5 Department of Medicine, University of British Columbia, Vancouver, BC V6Z 1Y6, Canada; 6 Department of Pathology and Laboratory Medicine, University of British Columbia, Vancouver, BC V6T 1Z7, Canada

**Keywords:** cardiovascular disease, drug repurposing, therapeutic target mapping, human specimens

## Abstract

**Background:** Although cardiovascular disease (CVD) significantly impacts the quality of life of millions of patients worldwide, the high costs of developing new drugs and conducting clinical trials for CVD hinder therapeutic development in this field. Repurposing approved drugs, of which the safety has already been tested can significantly reduce the time and cost required for treating CVD. From the perspective of pharmacology, repurposed drugs are selected to specifically target CVD patients carrying the matching drug targets, enabling tailored, personalized intervention. In the context of drug repurposing, therapeutic target mapping is a process used to assess the enrichment of molecules that can be affected by a drug in order to produce a therapeutic effect. **Methods:** This narrative review summarizes the workflows and challenges of performing therapeutic target mapping to assess the potential of repurposing existing drugs for treating CVD. We also share our perspective of how retrospective studies of human specimens can contribute to therapeutic target mapping based on experience from the Bruce McManus Cardiovascular Biobank (BMCB), a large explanted heart biobank located in Vancouver, Canada. The literature we discuss was identified by non-systematic searches of PubMed, using search terms “human-derived specimens”, “drug repurposing”, and “cardiovascular disease”. We reviewed articles from 2012 to 2026 and prioritized studies conducted after 2019 to discuss recent advances in the field. **Conclusions:** Human specimens of high molecular quality are needed for evaluating the enrichment of drug targets in CVD. Therapeutic target mapping in diseased cardiovascular tissue requires integrated expertise from medical, translational, and applied sciences to identify suitable human specimens for molecular phenotyping, and to design hypothesis-driven approaches to validate the drugs suggested for repurposing.

## 1. Introduction

In 2019 alone, worldwide 17.9 million people died from cardiovascular disease (CVD). Although CVD is the leading cause of death and hospitalization worldwide, the high cost of developing a new drug (an average of $2–3 billion USD) [1] and running clinical trials for CVD ($100–200 million USD for therapeutic agents approved by the Food and Drug Administration (FDA), from 2015 to 2016) [2] delay therapeutic development in this field. From 2018 to 2020, only 4 new drugs were approved by the FDA for cardiovascular indications (versus 49 for cancer) [3]. Repurposing approved drugs, of which the safety has already been tested in humans, can significantly reduce the time and cost investment needed to find novel therapeutics for cardiovascular indications. Drug repurposing strategies may arise from clinical observations, computational prediction, systems biology, transcriptomics, or target-based approaches. For example, in a 2012 clinical study, investigators observed that an anti-inflammatory drug (colchicine) used for gout also reduced the risk of heart attacks in gout patients [4]. The Colchicine Cardiovascular Outcomes Trial (COLCOT) trial then proved that colchicine lowered the risk of future ischemic cardiovascular events in patients who recently had a heart attack [5]. These findings led to several phase III clinical trials to test colchicine in patients with different CVDs, including myocardial infarction, chronic coronary artery disease, acute coronary syndrome, percutaneous coronary intervention, myocarditis, heart failure, and atrial fibrillation [6]. However, many of these trials failed to demonstrate efficacy [7,8]. This example demonstrates that repurposed drugs are not “one-size-fits-all”.

Drug repurposing clinical trials that have been rationalized by clinical observations have several disadvantages. First, these trials often study generic drugs rather than new compounds, making it difficult to attract industry support. Second, each study can only follow one drug in one disease sub-phenotype and results cannot be extrapolated to other sub-phenotypes. Lastly, the clinical endpoints designed for one disease trial may not provide adequate evidence to predict therapeutic effects in another disease. For example, the interleukin-1β monoclonal antibody canakinumab was tested in patients who recently had a heart attack in the Canakinumab Anti-inflammatory Thrombosis Outcome Study (CANTOS)and the treatment was also surprisingly associated with reduced occurrence of fatal and non-fatal lung cancers [9]. Two clinical trials were then launched to repurpose canakinumab for treating lung cancer and both failed to find any improved outcome [10,11]. Lung cancer was not the primary endpoint in the CANTOS trial, and so the time frame, number of lung cancer patients, and assessment of cancer risk were insufficient for a cancer trial. Moreover, no molecular evidence from the bench demonstrated that canakinumab is able to inhibit the growth of lung cancer cell lines [12]. The failed trials could have been avoided if this lack of evidence had been taken into consideration.

In the context of drug repurposing, therapeutic target mapping is a process used to assess the enrichment of molecules that can be affected by an existing drug. For any drug with known targets, the purpose of therapeutic target mapping is to confirm that these targets are also present in the new diseased tissue to be treated. Therapeutic targets are often drug receptors or enzymes in signaling pathways, which a drug act upon. To guide drug repurposing, therapeutic target mapping requires phenotyping of diseased cells or tissues to assess gene expression, protein abundance, as well as signaling pathway activity. The causal relationship between drug targets enriched in the tissue and disease progression must be investigated after therapeutic target mapping has been undertaken to predict treatment outcome.

This process is different from biomarker discovery and pharmacogenomics studies, which characterize indicators that are correlated with drug response, but that are not necessarily drug targets. Indeed, biomarkers and genetic makeup are often used for patient stratification, but not for identifying promising drugs for repositioning. By integrating molecular phenotyping with biobank-derived human tissues, therapeutic target mapping has the potential to substantially improve the precision and success rate of cardiovascular drug repurposing. This review provides examples of how existing drugs have been re-assessed for treating CVD and summarizes how biobanks can contribute to therapeutic target mapping, based on experience from the Bruce McManus Cardiovascular Biobank (BMCB), a large explanted heart biobank in Canada, and proposes strategies for predicting the efficacy of repurposed drug more precisely.

## 2. Therapeutic Target Mapping to Assess the Potential of Using Existing Drugs to Treat CVD

As an example of drug repurposing, rapamycin, a mammalian target of rapamycin (mTOR) inhibitor, was initially identified as an antifungal drug and immunosuppressant. It was successfully repurposed and is now used within drug-eluting stents to prevent restenosis after percutaneous coronary intervention (PCI) [13]. A therapeutic target mapping study found that mTOR activation in smooth muscle cells (SMCs) was upregulated in human arteries with restenosis, which confirmed the enrichment of targets of rapamycin in diseased vessels. Mechanistic study further validated the contribution of mTOR activation to the development of restenosis, and that rapamycin effectively downregulates genes involved in cell cycle transition to inhibit SMC proliferation [14]. This study demonstrated why mTOR inhibitors, but not any other cell growth inhibitors, were suitable for repurposing for this disease application. Canakinumab, a monoclonal antibody against interleukin-1β was originally approved to treat several autoinflammatory conditions, such as cryopyrin-associated periodic syndrome. Therapeutic target mapping demonstrated that the expression of the enzyme responsible for interleukin-1β production is increased in human atherosclerotic lesions [15]. This evidence in human specimens, as well as culture assays that confirmed the casual relationship between interleukin-1β and cell death, served as part of the biological basis for the CANTOS trial, which repurposed canakinumab to treat patients who recently had a heart attack; further, this work supported the inflammatory hypothesis of human atherosclerosis [16]. In these two examples, prior knowledge of drug pharmacology and risk factors for disease progression were needed to carry out therapeutic target mapping.

As an alternative, reverse transcriptome mapping has been developed as a systematic computational approach to screen existing drugs or compounds for repurposing. The principle is that if gene-expression signatures induced by drug A are inversely correlated with disease-associated gene-expression signatures induced by drug B, then drug A may be able to treat disease B [3]. This approach uses statistical models to assess the match between genes upregulated in disease and genes downregulated by drugs. Leveraging existing drug-transcriptome perturbation databases, such as L1000, computational methods can predict the efficacy of approximately 1000 drugs in a high-throughput fashion [17]. However, computational algorithms usually prioritize genes of statistical importance rather than disease-driving genes or pathways that can be therapeutically targeted. Predicted drug candidates can also vary based on the transcriptome databases used, and many of the signatures found in current databases were generated in cancer cell lines irrelevant to cardiovascular cells and disease [17,18]. Tanaka et al. adopted one of the statistical models, the Connectivity Map, to search for FDA-approved drugs that can inhibit a specific pathway involved in vascular calcification [19]. Among the 13 candidates selected by the computational method, only one, niclosamide, showed promising effects when tested in cultured human primary SMCs [19]. Therefore, there is a clear need to develop databases generated using CVD cell lines or human specimens to allow for reverse transcriptome mapping. Further, it is essential to validate in silico predictions and treatment outcomes using cell-based, biological CVD models.

When validating suggested drug candidates for repurposing, cell phenotypes that closely mimic those found in the diseased condition should be selected. Cells isolated from CVD tissues can maintain their phenotypes in early passages [20]. Human specimens and cells isolated from CVD tissues have been frequently used to determine molecular changes in response to disease stimuli, and drug treatment [18,21]. A recent study by Theodoris et al. identified a drug candidate for heart valve disease using a computational approach. They then cultured endothelial cells isolated from patient valves to validate that the drug candidate found can reverse the disease phenotype [22].

These examples and other recent work using therapeutic target mapping [19,22,23] illustrate the two commonly used workflows of drug repurposing for CVD. In the scenario of rapamycin and canakinumab, the therapeutic targets were well-known. The levels of these targets could be directly assessed by immunostaining, qPCR, or Western blot on diseased tissues to confirm their association with human disease progression. Then, functional assays using cultured cells or animal models could confirm the therapeutic effects. In the other scenario, when a promising drug has yet to be identified, patient-derived tissues and cells can be used to determine the molecules changed during disease progression. Computational approaches can then match the disease database with drug perturbation databases to identify promising drug candidates. Ultimately, the candidates need to be tested in cell and animal models to validate the in silico predictions. These workflows are straightforward, but why are they rarely implemented before launching clinical trials?

## 3. Challenges to Implementing Therapeutic Target Mapping

There are several challenges to implementing therapeutic target mapping. First of all, available human CVD specimens for therapeutic targeting mapping are limited and becoming even more so due to advancements in cardiac surgeries and interventions. Over the past 30 years, procedures have become less invasive, and shifted to surgeries that repair rather than replace diseased tissues. Aortic dissections and aortic aneurysms can be operated on via open surgeries to replace the damaged vessels using an artificial graft, or can be repaired by endovascular repair using a catheter to implant a stent graft. Since the adoption of drug-eluting stents, a 36% decreased in the rate of endarterectomy surgeries was seen in Ontario, Canada, between 2002 and 2014 [24]. This decrease was accompanied by an increase in carotid artery stenting [24]. In a study that followed 2502 patients who had undergone carotid endarterectomy surgery or carotid-artery stenting, the risk of stroke, myocardial infarction, or death were similarly low (2.5 years post-procedure) [25]. These data suggest that patients can be managed effectively by endovascular procedures without surgical removal of diseased specimens. Diagnosis using early-disease biopsies is also being replaced by blood biomarkers and advanced medical imaging. As samples become more limited, prospective biobanking for therapeutic target mapping will take more time to accumulate sufficient numbers of CVD specimens than before. A hidden effect of these changes in clinical guidelines and interventions is a potential bias in patient selection for therapeutic target mapping: only samples from those who are not suitable for endovascular procedures will be included in a study, and patients diagnosed by non-invasive methods at an early stage will be excluded. With continuing advances in early diagnosis and primary prevention, cardiovascular tissues collected at biobanks will consist of more end-stage disease samples, with the molecular profiles of these samples affected by disease progression, drug history, and comorbidities. An additional difficulty lies in the fact that information regarding disease trajectory, especially the molecular changes that occurred during disease onset, is often missing. For example, a viral infection can cause myocarditis and ultimately heart failure. Explanted hearts from these patients have already passed the stage of pathogen infection and are therefore not suitable for mapping anti-viral drugs. As another example, a study comparing coronary atherosclerotic lesions from patients with ischemic heart failure found that statin-treated patients had fewer lesions in the advanced stage [26]. Statins also changed the expression of genes involved in calcium regulation and the membrane repair machinery in biopsies of skeletal muscle [27], suggesting that drug history affects disease progression and molecular profile. These examples demonstrate that molecular profiles from end-stage CVD tissues have limited value for understanding the mechanism of disease onset and developing therapies for primary prevention. They are better suited for developing secondary or tertiary prevention strategies when residual risk factors can still drive disease progression despite primary intervention.

Moreover, finding the ideal “baseline control” is challenging. In mechanistic studies using cultured cells and genetically modified animal models, molecular changes along the disease trajectory can be investigated in different culture dishes or individual mice. Confounding factors can be well-controlled in the “baseline control” and “diseased” groups by using cells from the same batch and animals of the same strain and of similar age. Yet in human CVD studies, samples without CVD are often donated by younger adults (e.g., accidental death) who have not undergone long-term treatment (e.g., use of beta blockers) or experienced the comorbidities (e.g., diabetes and hypertension) seen in most older patients with CVD. These cofounding factors in “baseline controls” introduce molecular differences irrelevant to the disease trajectory. Sometimes, therapeutic target mapping can be done without the perfect “baseline controls”. For example, most studies of human atherosclerotic plaques are based on those removed by carotid endarterectomy surgeries, such as the Athero-Express study and the Biobank of Karolinska Endarterectomies study [28,29]. The Athero-Express study, initiated in 2002, has recruited more than 3000 participants undergoing carotid endarterectomy surgeries. Bulk RNA-sequencing of 654 advanced human carotid plaques revealed that the mTOR signaling pathway is enriched in patients with severe symptoms of transient ischemic attack or stroke [30]. This study suggested that it is feasible to assess the enrichment level of existing therapeutic targets (e.g., mTOR inhibitors) in clinically vulnerable patients without the use of healthy vessels. However, mTOR enrichment does not guarantee that mTOR inhibitors will alleviate the clinical symptoms of these patients.

Last but not least, there is a lack of motivation for pharmaceutical companies and clinicians to validate the therapeutic effects in pre-clinical models. Enrichment of a therapeutic target within the disease tissue means that the tissue will react to treatment, but the outcome is still unknown. These targets need to be disease-driving molecules to make sure that disease progression is inhibited or reversed following treatment. Therefore, therapeutic target mapping cannot replace mechanistic studies that are necessary to validate the causal relationship between targeted molecules and disease progression. The causal relationship is first confirmed in disease models in vitro. Using pharmacological (e.g., kinase inhibitors) or genetic approaches (e.g., knockdown), the activity of targeted molecules can be manipulated to determine their functional role in cell behavior. In vivo studies can further use genetic modification to change the activity of targeted molecules in animals (e.g., overexpression or knock-out) and assess the severity of disease. These rigorous mechanistic studies are important to predict therapeutic outcome. However, drug repurposing efforts often skips mechanistic validation studies. Taking the repurposing of colchicine as an example, the recently completed Colchicine in Percutaneous Coronary Intervention (COLCHICINE-PCI) trial [31] hypothesized that blocking the interleukin-1 pathway and the inflammasome pathway by colchicine will improve patient outcomes. Although pre-procedural administration of colchicine inhibited the surge of blood inflammatory biomarkers after PCI, this anti-inflammatory drug did not lead to reduced risk of PCI-related myocardial infarction and target vessel revascularization at 30 days [31]. These results suggest that although colchicine lowers blood inflammatory biomarkers, such as interleukin-6 and high-sensitivity C-reactive protein, it does not effectively lower the vascular inflammation caused by PCI-related injury. Whether the inflammatory pathways targeted by colchicine overlap with the inflammatory pathways of PCI-induced vascular injury is unknown. PCI is an endovascular procedure that does not remove any part of the diseased vessels. Most of our understanding about PCI-induced injury in humans is based on histology studies on autopsy samples [32,33]. Characterizing molecular pathways in stented vessels is challenging not only because post-mortem molecular degradation may introduce bias [34], but also because procedures used to remove the stent from the sample before tissue block embedding degrade RNA and proteins. Additionally, methods of inducing in-stent restenosis in small animals, including carotid artery ligation, cuff injury, and wire injury, do not fully recapitulate PCI-induced vascular injury [35]. Validating colchicine’s effects in a porcine model of PCI would require time and wet lab resources. Given that the safety of colchicine has already been tested in humans and its therapeutic effects in myocardial infarction have been validated, there is little incentive to re-validate its therapeutic effects in a similar CVD—especially when pre-clinical tests are expensive and time-consuming.

## 4. Examples of CVD Research at the Bruce McManus Cardiovascular Biobank

Biobanks that collect human-derived cells and tissues for research purposes play an important role in addressing the unmet needs in therapeutic target mapping. Retrospective studies of biobanked samples with high molecular quality can open the door for: (1) Developing molecular phenotyping methods to obtain human CVD molecular database; (2) Integrating clinical information to define “baseline controls”; and (3) Guiding the design of pre-clinical CVD models to validate the therapeutic effects of drug candidates.

The BMCB, initiated in 1982, has archived over 100,000 de-identified human cardiovascular tissue specimens, including explanted hearts from orthotopic heart transplantation, heart valves, and blood vessels. It is situated at the Centre for Heart Lung Innovation, a translational research centre within Providence Health Care’s St. Paul’s Hospital, a teaching hospital affiliated with the University of British Columbia. The BMCB follows a hybrid model of general biobanking for future research and fit-for-purpose biobanking for specific studies. The long-term vision of general biobanking for future research is to enable the rapid launch of retrospective studies. Rare human specimens are collected for a wide spectrum of molecular phenotyping applications, including bulk RNA-sequencing, proteomics, single-nucleus sequencing, multiplex imaging, and spatial transcriptomics.

**Figure 1:**
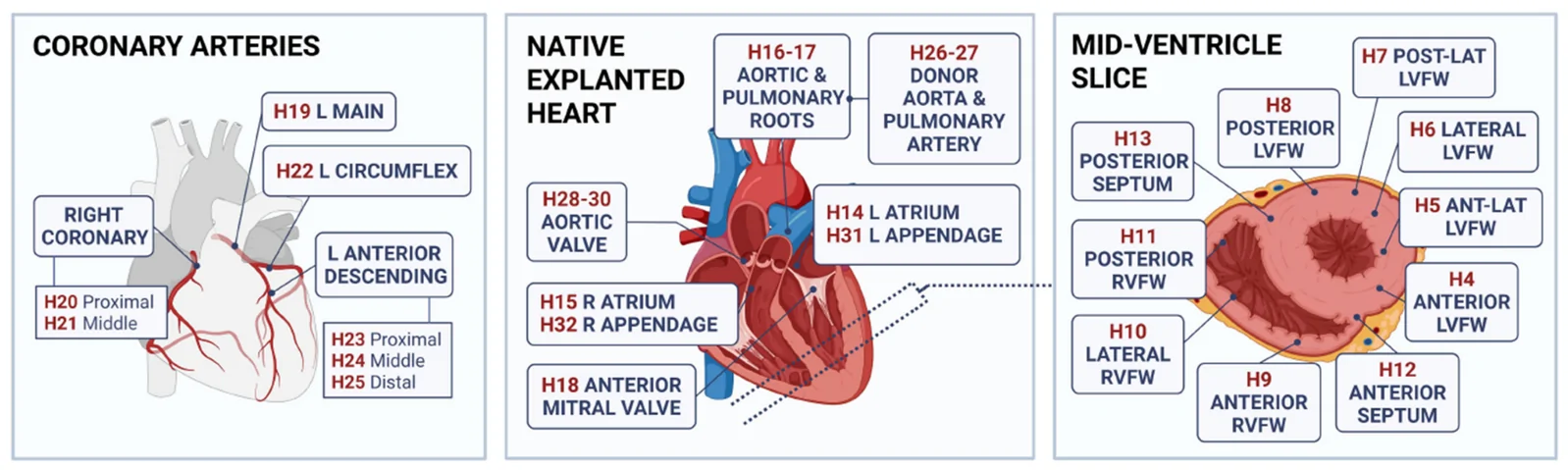
**Standardized biobanking regions of explanted hearts.** 29 segments are collected at standardized anatomical locations from each heart. Each segment, or H#, is designated a standard number corresponding to its specific anatomical region for consistent biobanking. Every segment is further divided into four pieces and archived in four formats: flash-frozen, formalin-fixed paraffin embedded (FFPE) blocks, optimal cutting temperature (OCT) compound blocks, and RNAlater-preserved to ensure comprehensive preservation for multipurpose translational research. L.: left; R.: right; Ant: anterior; Post: posterior; Lat: lateral; LVFW: left ventricular free wall; RVFW: right ventricular free wall.

The signature collection at the BMCB is the explanted hearts of heart failure patients. St. Paul’s Hospital conducts adult heart transplants for the entire province of British Columbia, acting as a hub for patient recruitment and collection of specimens affiliated with clinical information. The explanted hearts and clinical information are consented and de-identified for future research. Following surgical explant, hearts are immediately immersed in a cold preservation buffer and transported from the operating room to the biobank within 15 min. Each heart has 29 segments collected at standard locations (Figure 1), and every segment is divided into four pieces to be archived in different formats: (1) flash-frozen, (2) formalin-fixed paraffin-embedded (FFPE), (3) optimal cutting temperature (OCT) compound-embedded, and (4) RNAlater-preserved. The availability of structure-preserved formats (e.g., FFPE) for each segment enables histopathological classification for sample selection, whereas the clinical information of each patient is used to select the patient cohort.

**Figure 2:**
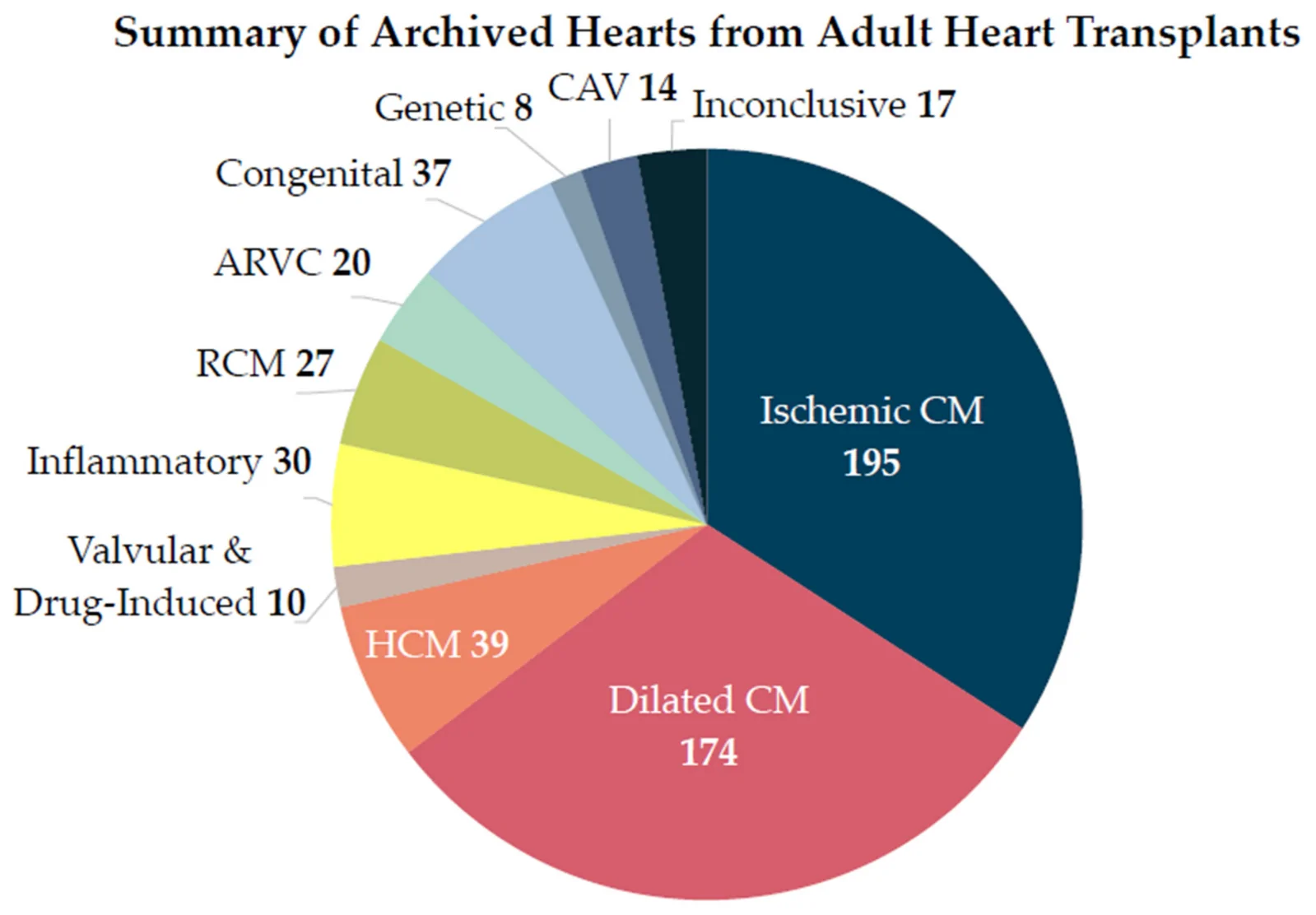
Archived explanted hearts at BMCB, categorized by primary diagnosis. The BMCB currently houses 571 failed hearts from adult orthotopic heart transplants (as of 1 November 2025). Primary diagnosis was determined based on clinical and pathological criteria. In the cases of coexisting etiologies, explanted hearts were categorized under a single diagnosis based on the main underlying cause of heart failure. Numbers indicate hearts per diagnosis. F: female (*n* = 160); M: male (*n* = 411); CM: cardiomyopathy; CAV: cardiac allograft vasculopathy; ARVC: arrhythmogenic right ventricular cardiomyopathy; RCM: restrictive cardiomyopathy; HCM: hypertrophic cardiomyopathy. *Inflammatory diseases* include myocarditis (lymphocytic, granulomatous, eosinophilic, viral, rheumatic, and giant cell). *Congenital diseases* include transposition of the greater arteries, tetralogy of Fallot, congenital non-compaction, Ebstein anomaly. *Genetic diseases* include mitochondrial cardiomyopathy, Marfan syndrome, Becker muscular dystrophy, Danon disease, and glycogen storage disorders. *Inconclusive* refers to cases where there was insufficient pathological information to provide a definitive diagnosis.

Investigators with human ethics approval or exemption to investigate de-identified human specimens in their local research institutions can submit a request to the biobank with a research summary specifying how the human specimens will be characterized. Each request is assessed by the biobank’s Scientific Committee based on the scientific merit of the research topic, feasibility of using the proposed procedures to obtain meaningful deliverables, and risk of affecting patient privacy (e.g., genetic testing). Studies that have the potential to impact a broad health and research community are prioritized and allowed access to use these precious samples for research purposes. In the past 30 years, the BMCB has collected 571 failed hearts (Figure 2), offering an unparalleled resource for generating a human CVD molecular database that can be used to advance drug repurposing. Several examples of how researchers have used BMCB samples to surmount challenges in therapeutic target mapping are detailed below.

### 4.1. Developing Protocols and Quality Standards for High-Throughput Molecular Phenotyping of Archived Specimens

Since it can take years to accumulate adequate sample sizes when prospectively biobanking, archived samples can be leveraged and the output of molecular phenotyping increased using high-throughput ‘Omics’ technologies. Developing protocols and quality assessment standards for different biobanking formats (e.g., flash-frozen and FFPE) is critical for generating a high-quality molecular database for therapeutic target mapping. Whole transcriptome sequencing of atrial appendages observed increased degradation and proportions of off-target intergenic and intronic reads in samples preserved in FFPE blocks compared to those in frozen samples [36]. RNAlater-preserved rat hearts have a lower RNA yield compared to snap-frozen samples [37]. Single-nucleus RNA sequencing and spatial transcriptomics are new ‘Omics’ technologies that obtain whole transcriptome information from flash-frozen tissues and tissue blocks. We have developed a protocol to tackle the long-standing technical issue of dissociating intact nuclei from flash-frozen cardiac muscles in explanted hearts [38]. These nuclei can be used for single-nucleus RNA sequencing to understand the heterogeneity of cell status, cellular function, and signaling pathways in failed hearts. To select high-quality samples for spatial transcriptomics, DV200, which evaluates the percentage of fragments of >200 nucleotides, used to be the gold standard method of assessing RNA integrity in samples archived in FFPE blocks. However, we have demonstrated that DV200 alone cannot predict RNA degradation in samples from warm autopsies and we recommend combining DV200 with RNA yield to assess sample quality in order to generate high-quality spatial transcriptomics data [34]. With the help of these protocols and quality standards, the generated ‘Omics’ database can be used in different workflows of drug repurposing—either with known therapeutic targets or to enable in silico predictions. Developing high-throughput molecular phenotyping of archived samples can mitigate the slow recruitment of prospective samples for individual studies as one sample can support research investigating multiple different targets.

### 4.2. Integrating Pathology to Define “Baseline Controls”

As shown in Figure 1, 29 segments are collected from standard locations in an explanted heart regardless of pathological classification. Therefore, coronary arteries or myocardium from different locations in one explanted heart can be at various disease stages according to pathological definition. In a failed heart with ischemic cardiomyopathy, the BMCB archives seven segments of coronary arteries from different locations (Figure 1, H19-H25). Of these seven segments, one segment can be at an early stage of atherosclerosis (e.g., fatty streak or pathologic intimal thickening), while the other segments from the same patient may contain advanced fibroatheromas [39]. We can use samples at pre-clinical stage or early disease stage as “baseline controls” to assess the enrichment of targets in advanced disease [40]. In one tissue section, different cells and tissue compartments may not be affected by disease equally. Based on histology, pathologists can define the least affected regions as “baseline controls” for the diseased regions. For example, SMCs in the tunica media are less affected during atherogenesis compared to those in the tunica intima. Using high-throughput spatial transcriptomics, we have recently characterized the molecular changes in intimal SMCs using medial SMCs as “baseline controls” in the same section of coronary artery with diffuse intimal thickening (DIT).

Unlike mice, which are frequently used as atherosclerosis models, humans develop DIT in the coronary arteries at as early as 36 weeks of gestation [41]. In DIT, the thickened intima layer is enriched with SMCs like the medial layer [42]. DIT is considered to be an adaptive response to mechanical stresses [43] and a non-pathological condition [44]. However, the locations of DIT in early life overlap with those of atherosclerosis in later life [41], suggesting that SMCs in the DIT promote disease onset more than SMCs in the medial layer. We used spatial transcriptomics to explore the molecular differences between the SMCs in the two layers and compared DIT SMCs with those in atherosclerotic lesions [34]. Although DIT SMCs had switched to a synthetic state compared to contractile SMCs in the media, they did not exhibit the chondrogenic, osteogenic, and proinflammatory features of lesion SMCs [45]. Therefore, DIT SMCs are at a “primed,” but stable status [34]. This molecular evidence supports the current pathological classification of DIT as a pre-clinical stage of atherosclerosis [34]. Moreover, it showcases the feasibility of integrating pathology to define “baseline controls” and to study molecular changes along the disease trajectory.

### 4.3. Guiding Pre-Clinical Models to Validate Therapeutic Effects of Drug Candidates

Pre-clinical models for drug screening are meant to recapitulate the disease pathobiology seen in humans. Hence, understanding the molecular changes in cells being affected by human CVD can guide the development of pre-clinical models to validate the therapeutic effects of drug candidates. The traditional dogma of coronary artery disease is that it is an immune-driven response to injury due to lipid deposition in the vessel walls. This dogma was overturned using samples from the BMCB. Allahverdian et al. used a modified lipid staining method to distinguish intracellular lipids from those in the extracellular matrix of the coronary lesions [46]. Approximately half of the lipid-loaded foam cells were found to be SMCs instead of macrophages [46]. Using multiplex imaging and spatial analysis of human coronary lesions, Elishaev et al. confirmed the theory that lipid deposition starts in the deep intima where SMCs are enriched and readily available to take up cholesterol to form foam cells [39]. These two studies revealed the underestimated role of SMCs in initiating coronary artery disease, which was previously attributed to immune cells. Cholesterol efflux from SMCs is more difficult than from macrophages because SMCs do not have an efficient machinery to metabolize and mobilize the cholesterol [46,47,48]. Therefore, pre-clinical models for testing lipid-lowering drugs should not only consider macrophage-derived foam cells, but also SMCs.

These examples showcase how retrospective studies of archived human CVD can advance therapeutic target mapping: (1) high-throughput ‘Omics’ technologies on archived samples can be used to obtain high-quality molecular data; (2) multiple samples from the same patient or different regions of one sample can be used to identify molecular changes along the disease trajectory; and (3) samples can advance understanding of disease pathobiology and guide the design of new pre-clinical drug testing models. The examples also illustrate the various areas of expertise required to advance therapeutic target mapping: clinical sciences, translational sciences, and applied sciences (e.g., computational methods).

## 5. Integrating Expertise to Advance Therapeutic Target Mapping

The integration of clinical sciences and translational sciences is essential for selecting the right human specimens for therapeutic target mapping. Explanted hearts primarily represent advanced disease, although they may contain tissue regions corresponding to earlier pathological stages. These explanted hearts are usually collected from patients who have already undergone primary interventions, such as treatment with lipid-lowering drugs. The molecular profiles from these human specimens describe biological alterations that may have resulted from primary interventions; these profiles can thus support therapeutic target mapping to identify secondary prevention drugs. Taking anti-inflammatory drugs as an example again, colchicine was repurposed for secondary prevention in patients who had chronic coronary disease (Low-Dose Colchicine, LoDoCo trial) [49]. All patients recruited in this clinical trial had received statins. Pre-clinical studies have shown that statins have direct anti-inflammatory effects and inhibit the NFκB signaling pathway [50]. If therapeutic target mapping is performed using specimens from patients who have not received statins in the past, it is likely that the anti-inflammatory effects of colchicine for secondary prevention will be overestimated. Therefore, clinical history, pathological features, and knowledge of molecular biology and pharmacology must all be integrated in order to critically select human specimens for therapeutic target mapping.

Computational analysis pipelines have focused on combining information about disease etiology with that from CVD-specific databases to identify disease-relevant targets instead of statistically important targets. Differentially expressed genes are statistically important targets in transcriptomic analysis. However, their upstream regulators, such as transcription factors, may not change at the mRNA level during disease progression. Relying on reverse transcriptome mapping alone may miss these potential disease-driving targets [51]. Regulatory network analysis is frequently combined with single-cell RNA sequencing (scRNA-seq) and Assay for Transposase-Accessible Chromatin with sequencing (ATAC-seq) to identify transcription factors that drive the cell phenotypes seen in CVD [45,52,53]. These studies use prior knowledge of system biology and apply CVD-specific databases generated by human specimens or animal models to screen for potential master regulators. Pseudo-trajectory analysis leverages heterogeneity in cell status within the tissues to reconstruct cellular changes during disease progression. This analysis is commonly adopted to mitigate the limitation of characterizing end-stage human CVD tissues, which represent a snapshot of disease outcome. Several recent studies applied cell-type-specific ‘Omics’ to CVD samples of various pathological stages and used the ‘Omics’ data to validate results from pseudo-trajectory analysis [54,55,56]. These studies advanced our understanding of cell phenotypes and genes that are strongly correlated with different stages of CVD. Analysis pipelines, such as MAGMA and LDSC-SEG, can further assess the contribution of these disease-correlated changes to genetic traits of CVD [54,57].

In recent years, most biobanks have started collecting paired blood and tissue samples. Correlation studies of molecular profiles between blood and diseased tissues will enable personalized medicine using blood biomarkers to identify patients who will benefit from repurposed drugs. Besides computational sciences, bioengineering expertise can advance the design of pre-clinical models for validating therapeutic effects. For example, porcine coronary arteries can be cultured ex vivo to assess anti-restenosis effects [58]. Cultured organoids derived from induced pluripotent stem cells (iPSCs) may be a new strategy to develop humanized models for validating repurposed drugs. Human iPSC-derived cardiomyocytes can be used to form organoids ex vivo to mimic the heart ventricles [59]. In a recent study, computational approaches identified bosutinib as a promising drug candidate for venous malformation and its therapeutic effects were validated in ex vivo cultured blood vessels [23]. The dilated blood vessel model used in this study was created using genetically modified venous endothelial cells derived from iPSC [23].

## 6. Conclusions and Future Directions

We conclude that therapeutic target mapping is a logical stepping stone to facilitate drug repurposing for different CVD subgroups and progression to clinical trials. We therefore propose five strategies for biobanks to adopt to advance drug repurposing:

(1) *Creating broad consent mechanisms for future research so that archived specimens can be used for therapeutic target mapping by different workflows and researchers.*
  The biobank can have a centralized consent form that includes ethics for collecting and archiving human specimens for unspecified future research. The biobank needs to consult with the research ethics board to phrase the terms in the consent form, ensuring that patient privacy and voluntary participation will be guaranteed, permission for use by multiple research projects is clearly explained, and the logistics of material, data, and intellectual property transfers are considered.
(2) *Developing high-throughput and multi-dimensional molecular phenotyping technologies to reveal a broad spectrum of potential therapeutic targets in CVD tissues, allowing target mapping for any existing drugs or compounds being investigated by industry.*
  Biobanks need to obtain feedback from users and test the quality of their archived samples regularly. The quality control standards used need to ensure that sample quality meets the requirements of phenotyping technologies, allowing the biobank to assess the compatibility of samples with downstream assays and make timely updates to the sample preservation protocols.
(3) *Benchmarking and tailoring computational methods to integrate CVD molecular databases with clinical databases, enabling in silico therapeutic target mapping in a high-throughput fashion.*
  Computational methods often prioritize statistically important targets, which can be skewed by variance in sample handling and reporting bias in clinical databases. Knowing how the input data has been generated is critical for interpreting and validating the output data of any computational pipeline. Therefore, a biobank can serve as a consultant in benchmarking and tailoring computational methods.
(4) *Leveraging patient-derived cells and specimens to provide CVD-relevant in vitro models for drug/compound screening and validation of results from in silico therapeutic target mapping.*
  Biobanks can develop protocols for isolating and preserving primary cells together with researchers, enabling future use in cell characterization and culture studies.
(5) *Aligning investigation of CVD tissues, blood samples, and medical imaging from the same patients to advance biomarker discovery and clinical diagnosis, as well as providing information to guide patient stratification in future clinical trials.*
  This strategy requires information sharing among hospitals, biobanks, and research facilities. Building an infrastructure that supports data storage and sharing is challenging because data systems differ between institutions and units. Biobanks should seek support from the government, industry, and the general public to enable information sharing and maintain operational sustainability.

In addition to being the centralized repository of clinical resources, biobanks need to develop in-house expertise to guide the design of biobanking and sample selection. This in-house expertise is built upon knowledge exchange with research users, including surgeons, cardiologists, pathologists, and scientists in different fields. Given that it often takes many years to accumulate an adequate amount of CVD samples for research, biobanks need to have a long-term plan for their sustainability and knowledge translation. They must start by creating synergy among researcher users in clinical, translational, and applied sciences, as well as industry partners.

## Data Availability

No new data were created or analyzed in this study. Data sharing is not applicable to this article.

## Abbreviations

The following abbreviations are used in this manuscript:

BMCB	Bruce McManus Cardiovascular Biobank
CANTOS	Canakinumab Anti-inflammatory Thrombosis Outcome Study
CVD	cardiovascular disease
COLCHICINE-PCI	Colchicine in Percutaneous Coronary Intervention
COLCOT	Colchicine Cardiovascular Outcomes Trial
DIT	diffuse intimal thickening
FDA	Food and Drug Administration
FFPE	formalin-fixed paraffin-embedded
iPSCs	induced pluripotent stem cels
LoDoCo	low-dose colchicine
mTOR	mammalian target of rapamycin
OCT	optimal cutting temperature
PCI	percutaneous coronary intervention
SMC	smooth muscle cells
